# Construction, analysis and validation of co-expression network to understand stress adaptation in *Deinococcus radiodurans* R1

**DOI:** 10.1371/journal.pone.0234721

**Published:** 2020-06-24

**Authors:** Suraj R. Joshi, Surabhi Jagtap, Bhakti Basu, Deepti D. Deobagkar, Payel Ghosh

**Affiliations:** 1 Bioinformatics Centre, Savitribai Phule Pune University, Pune, India; 2 Molecular Biology Research Laboratory, Department of Zoology, Savitribai Phule Pune University, Pune, India; 3 Molecular Biology Division, Bhabha Atomic Research Centre, Mumbai, India; Universidade Federal de Santa Maria, BRAZIL

## Abstract

Systems biology based approaches have been effectively utilized to mine high throughput data. In the current study, we have performed system-level analysis for *Deinococcus radiodurans* R1 by constructing a gene co-expression network based on several microarray datasets available in the public domain. This condition-independent network was constructed by Weighted Gene Co-expression Network Analysis (WGCNA) with 61 microarray samples from 9 different experimental conditions. We identified 13 co-expressed modules, of which, 11 showed functional enrichments of one or more pathway/s or biological process. Comparative analysis of differentially expressed genes and proteins from radiation and desiccation stress studies with our co-expressed modules revealed the association of cyan with radiation response. Interestingly, two modules viz darkgreen and tan was associated with radiation as well as desiccation stress responses. The functional analysis of these modules showed enrichment of pathways important for adaptation of radiation or desiccation stress. To decipher the regulatory roles of these stress responsive modules, we identified transcription factors (TFs) and then calculated a Biweight mid correlation between modules hub gene and the identified TFs. We obtained 7 TFs for radiation and desiccation responsive modules. The expressions of 3 TFs were validated in response to gamma radiation using qRT-PCR. Along with the TFs, selected close neighbor genes of two important TFs, viz., DR_0997 (CRP) and DR_2287 (AsnC family transcriptional regulator) in the darkgreen module were also validated. In our network, among 13 hub genes associated with 13 modules, the functionality of 5 hub genes which are annotated as hypothetical proteins (hypothetical hub genes) in *D*. *radiodurans* genome has been revealed. Overall the study provided a better insight of pathways and regulators associated with relevant DNA damaging stress response in *D*. *radiodurans*.

## Introduction

The *Deinococcaceae* family bacterium *D*. *radiodurans* R1 is a gram-positive coccus that withstands several stress conditions such as gamma radiation, UV radiation, desiccation and various other clastogens like methyl methanesulfonate, *N*-methyl-*N*-nitro-*N*-nitrosoguanidine (MNNG), nitrous acid, and hydroxylamine [1, 2]. The organism sustains nearly 200 DNA double-stranded breaks owing to highly efficient sequential DNA repair pathways, highly expressed enzymatic and non-enzymatic processes for cellular detoxification and compact genome structure etc. [3]. The genome of *D*. *radiodurans* comprises of two chromosomes along with two plasmids corresponding to 3.3Mb genome size. The genome comprises of 3,212 genes, of which 3,079 correspond to protein-coding genes, while, functionality for 1,468 genes is still unknown [4, 5]. Since the last six decades, researchers have employed various approaches to gain meaningful insights into the mechanisms responsible for radiation and desiccation resistance in *D*. *radiodurans* [6, 7].

The functional characterization of genes and regulators has been worked out in the context of network analysis [8]. WGCNA approach is one of the promising tools for studying the functionality of uncharacterized or hypothetical proteins [9, 10]. This methodology has been extensively utilized in various organisms for predicting biological functions of gene/s in a cluster [9–15]. Moreover, this approach has been applied in constructing a co-expression network using a diverse set of conditions or data sets [16, 17].

Condition dependent (radiation) network has already been reported for *D*. *radiodurans* using Bayesian approach which revealed various pathways important for radiation stress adaptation [18]. However, the study does not encompass possible regulators of pathways involved in stress adaptation. Here we report a condition-independent coexpression network for *D*. *radiodurans* detailing pathways and regulators involved in radiation stress adaptation.

Eleven co-expressed modules out of thirteen showed significant enrichment of 28 KEGG pathways, 173 biological processes and 31 molecular functions. Genes clustered in a module identified through WGCNA represent association with the same biological function [19]. The coexpressed modules, which are found to be associated with radiation and desiccation response, were further analyzed for their biological significance/s. TFs mapped in a module were obtained from Predicted Prokaryotic Transcription Factors (P2TF) database [20]. Expression of selected TFs and their close neighbors were validated using qRT-PCR in response to gamma radiation stress. These close neighbors were selected based on the current WGCNA network as well as using the previous literature [21].

To summarize, the present study revealed regulators and pathways associated with radiation and desiccation stress. Based on the correlation of TFs with the hub gene, our analysis indicated that genes involved in DNA repair and metabolism, amino acid, benzoate degradation, quorum sensing, oxidoreductase, ATPase, endonuclease activity, translation and transport etc. could be regulated by DR_0997 (Transcriptional regulator, FNR/CRP family), DR_A0071 (transcription repressor SmtB) or DR_2287 (AsnC family transcriptional regulator). Besides this, the guilt by association and Topological Overlap Measure (TOM) approach [8] was used to assign functionality to 5 hypothetical hub genes. Hence, it is indicated that different stress responsive pathways and TFs may act in a concert, for the survival of *D*. *radiodurans* under stress.

## Materials & methods

### Microarray data acquisition

Microarray data sets available for *D*. *radiodurans* R1 under diverse stress conditions were downloaded from NCBI-GEO database [22, 23]. The datasets were double-channelled, pre-normalized by LOWESS method and background corrected. All the samples were merged into a single file, where rows are genes and columns were samples. The detailed data frame and R scripts used to generate the results are provided in supplementary files S1.zip and S2.zip respectively. The data sets comprised of 9 experimental conditions corresponding to 61 microarray samples, together consisting of 3163 probe sets. The microarray experiments used in this study were indicated with NCBI GEO accession numbers as follows: GSE59138, GSE59135, GSE18661, GSE33758, GSE17720, GSE17722, GSE17724, GSE20383 and GSE29516 (S1 Table). The overall distribution of microarray data was assessed by a boxplot. Also, the extent of batch effect, if any was evaluated by MDS (Multidimensional scaling) based analysis of the different datasets. Such dimensional reduction techniques have been reported in several previous works [24–26] towards evaluating the presence/extent of batch-effects. The plot was obtained for MDS analysis using PAST program [27].

### Construction of co-expression network and assessment of module stability

The WGCNA R package was used for network construction, module detection and hub gene selection [9]. Gene expression profiles across diverse experimental conditions were used to calculate the Biweight mid correlation (bicor). This function is a good alternative to Pearson correlation coefficient since it is more robust to outliers [28]. Using the absolute values of correlation, the similarity matrix was obtained by S_ij_ = | cor (i, j)|, where cor (i, j) is the correlation of gene_i_ and gene_j_. The similarity matrix was converted to the adjacency matrix. The adjacency matrix (AdjMat_ij_ = |S_ij_|^β^) was computed by raising the similarity to the power β, using the scale-free topology criterion. We selected the power β for which the scale-free topology fitting index (R^2^) was 0.9, by plotting R^2^ against soft thresholds. For this study, a soft-threshold power (β) of 6 was used. The weighted adjacency matrix was transformed into a TOM matrix and the corresponding dissimilarity was calculated. The defined values of the dissimilarity matrix were used as an input for hierarchical clustering and the modules were identified in the resulting dendrogram by applying dynamic hybrid tree cutting algorithm. Finally, the modules with highly correlated Module Eigengenes (ME > 0.8) were merged. The minimum height for merging, the modules were set to 0.2 which corresponds to the correlation value of 0.8. The reproducibility of modules was tested by using random sampling test as described earlier [17]. Briefly, half of the microarray data i.e. 30 out of 61 microarray samples were randomly sampled 1000 times to obtain the modules. The connectivity correlations between the original module and the one obtained after 1000 times random sampling were calculated and expressed as the Mean ± SD. The module was considered as stable when connectivity correlation was > 0.7.

### Hub gene identification and annotation

Hub genes were identified by considering three factors: K_extracted_ (node degree), K_within_ (sum of adjacencies of connected genes in extracted modules) and the correlation with ME (>0.8) were selected as mentioned previously [17]. We chose the top hub genes from each module using “chooseTopHubInEachModule” function of WGCNA. Functionality was assigned to hypothetical hub genes based on gene ontology of close neighbors of hub genes (TOM > 0.01) as described earlier [8].

### Network visualization and functional analysis

Intramodular connections were extracted using WGCNA “exportNetworkToCytoscape” function and visualized in Cytoscape 3.5.1 [29]. To understand the potential role of these modules the gene ontology enrichment analysis was performed using ClueGO, a widely used Cytoscape 3.5.1 plugin [30]. A two-sided (Enrichment/Depletion) test based on hypergeometric distribution was applied to get the statistically significant p value of the gene ontology analysis.

### Identification of stress responsive module and transcription factors through comparative analysis

Differentially Expressed Genes (DEGs) [21] and proteins (DEPs) [31] from post-irradiation and DEGs [32] and DEPs [33] from post desiccation recovery experiments were mapped with respect to all 13 modules to obtain significant radiation and desiccation associated modules. This is to be noted that the experiments from which the above DEGs and DEPs were used for comparison, were omitted from the construction of the co-expression network. To infer the statistical significance (*p* value ≤ 0.05) of the predicted radiation and desiccation associated modules, we used a hypergeometric distribution test. We also mapped the TFs of *D*. *radiodurans* into the predicted radiation and desiccation stress associated modules. TFs were obtained from P2TF (Predicted Prokaryotic Transcription Factors) database that contains 372,877 TFs from 1,987 prokaryotes [20]. TFs are ranked based on the correlation of TFs with module hub gene [34–36]. The TFs thus obtained in these modules were further confirmed for the presence of DNA binding and other regulatory domain using Conserved Domain Database (CDD) [37].

### Validation of regulators in response to radiation stress by qRT-PCR

Wild type (WT) *D*. *radiodurans* was grown in TGY (1% bactotryptone, 0.1% glucose, and 0.5% yeast extract) broth at 32°C overnight and irradiated at sublethal dose of gamma radiation (Dose = 6 kGy, Dose rate = 1.8kGy/hour, Model = Gamma Cell 5000, Bhabha Atomic Research Centre) as described earlier [31]. Post-irradiation recovery (PIR) was carried out for 90 minutes under standard growth conditions. RNA was extracted after 90 minutes of PIR using Direct-Zol^TM^ RNA MiniPrep kit (Zymo Research USA) as per manufacturer’s protocol. Genomic DNA was eliminated using the TURBO DNA free kit (Life Technologies, USA) according to the manufacturer’s protocol. The cDNA was synthesized using 1μg of input RNA by Applied Biosystems™ High-Capacity cDNA Reverse Transcription kit as per manufacturer’s instructions. Expression profile of selected TFs in a module or their co-expressed genes were assessed using SYBR Green master mix (TAKARA, USA) and CFX96 Touch Real-Time PCR Detection System (Bio-Rad, USA) in response to gamma radiation stress. List of oligo-nucleotides for qRT-PCR is given in S2 Table. Three biological replicates were included for each irradiated or un-irradiated conditions. 16S rRNA was used as an internal reference gene. Student’s *t*-test was applied to infer the statistical significance (*p* value ≤ 0.05) of the data using GraphPad Prism 5.0 (GraphPad Software, Inc., La Jolla, CA, USA).

## Results and discussion

### Microarray data sets used for building co-expression network

The condition-independent network constructed in the present study included diverse data sets generated to compare either effect of knockout mutation or various abiotic stresses on *D*. *radiodurans* by comparing transcriptomes of stressed and control cells. The brief description and outcome of these experiments [38–44] are provided in S3 Table. Boxplot (S1A Fig) suggest that the overall distribution of microarray samples is similar. No discernable batch-effect was indicated from MDS analysis and the plot shows a single cluster of most of the data points/ samples considered in this study (S1B Fig). Combining these diverse datasets for condition-independent network allowed us to (i) study the gene pairs that are expressed at the same time irrespective of the change in conditions, and (ii) reveal the potential regulators important for stress adaptation of the organism. Therefore, we hypothesized that the co-expressed links or genes obtained will be either evolutionarily conserved or regulated by the same TF or may form a part of same biological process.

### Construction of coexpression network and module identification

**Fig 1:**
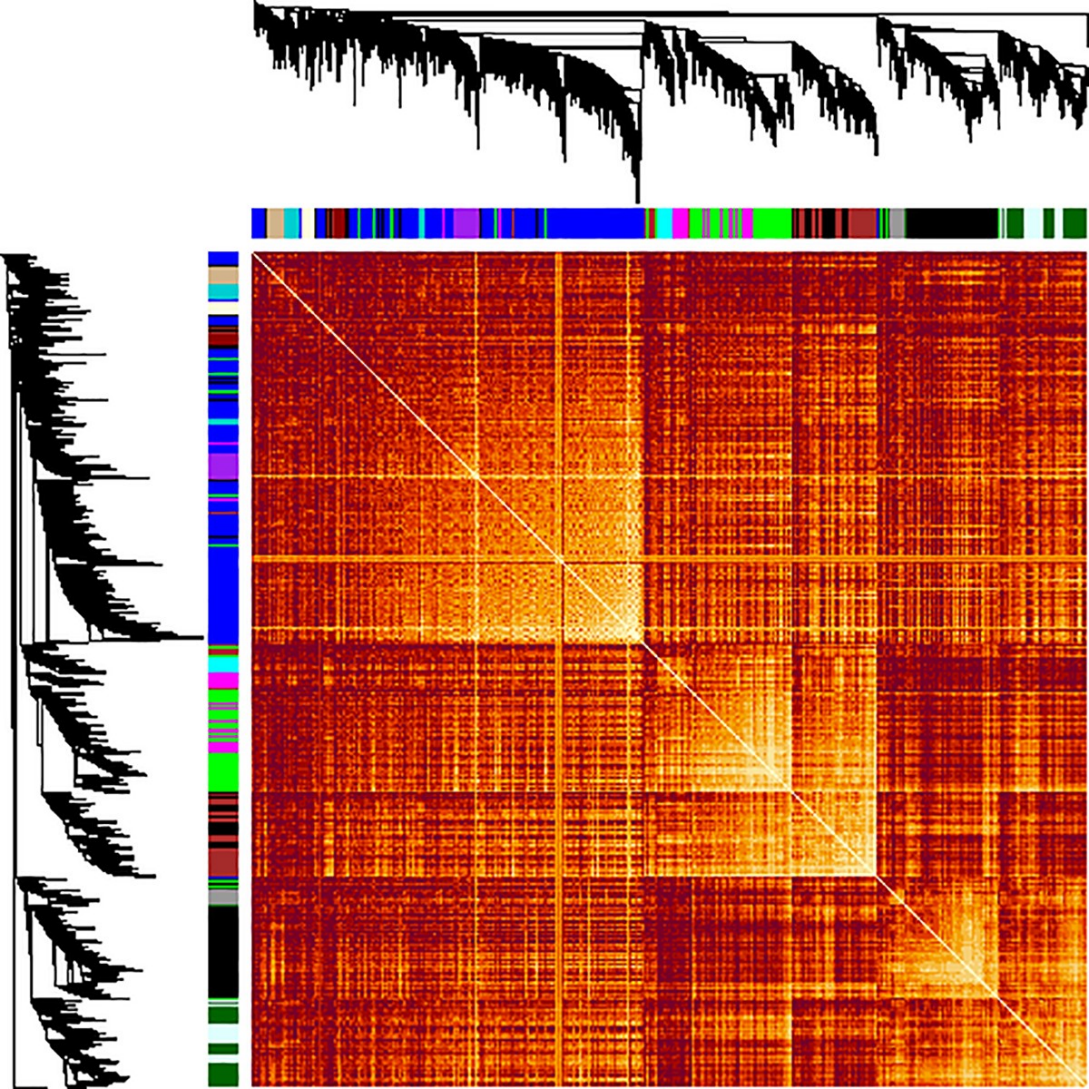
Topological overlap matrix plot. A topological overlap matrix (TOM) of the weighted gene co-expression network consisting of ~3163 genes. The branches on the left side of the plot indicate gene dendrogram and top side indicates module assignment. Different color bars are displayed below and right side of the dendrogram represents a module. The light color boxes along the diagonal represent a module.

**Fig 2:**
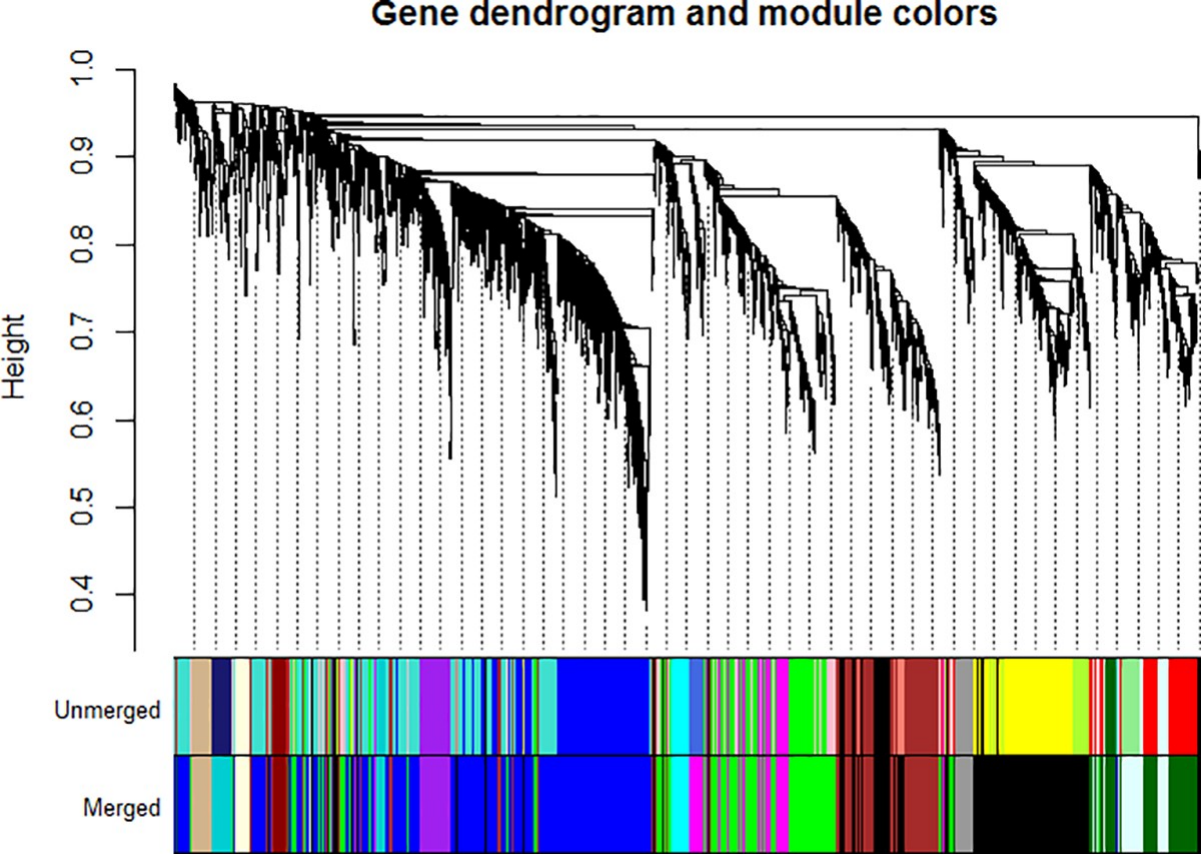
Gene dendrogram and module color. Cluster dendrogram is based on the topological overlap of gene expression across diverse conditions. The coloured panel beneath the dendrogram shows modules obtained from WGCNA study. The upper panel indicates unmerged modules, whereas the lower panel shows modules after merging based on ME values. Each module possesses at least 20 genes.

**Fig 3:**
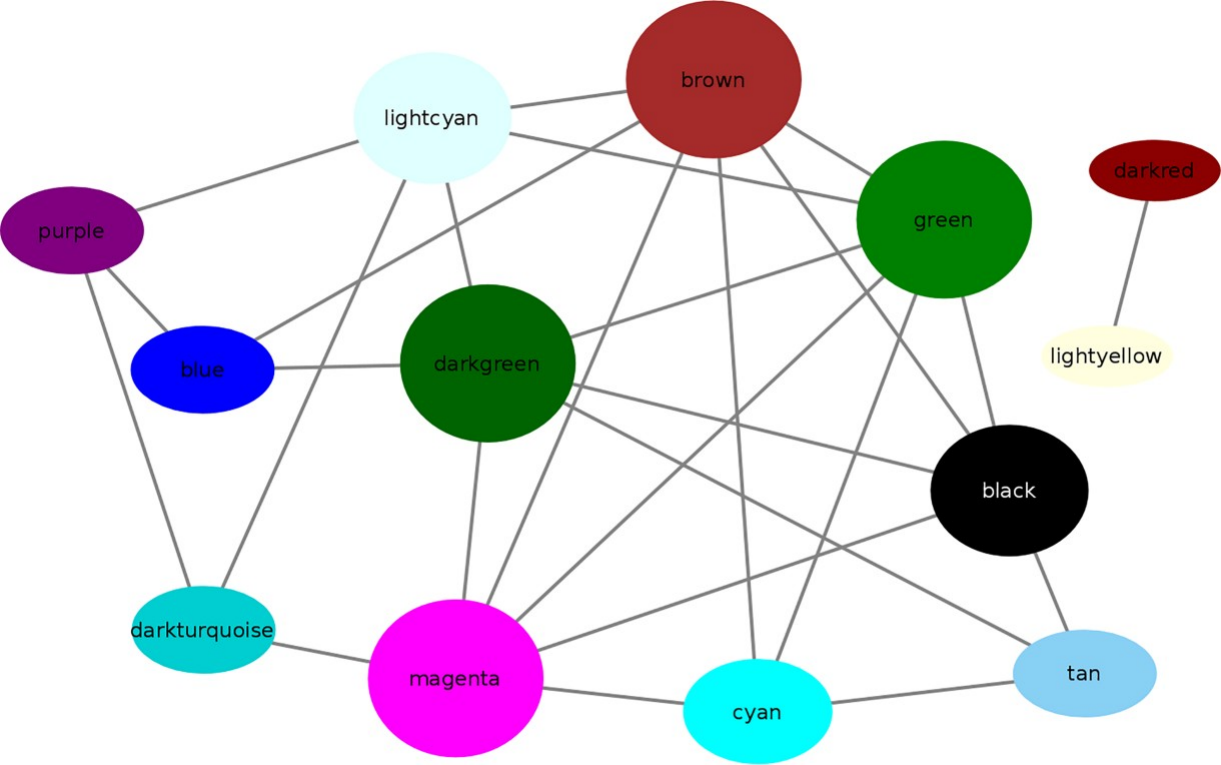
Inter-modular connectivity. Inter-modular connectivity is calculated based on ME. Larger size modules have a high degree (more connections), whereas smaller size modules depict fewer connections with other modules in a network. Modules like darkgreen, brown, green, magenta, black, lightcyan, cyan, tan, blue, purple and darkturquoise showed high degree, whereas smaller modules like darkred as well as light yellow showed a low degree or fewer connections. The edges correspond to ME value between the modules.

**Fig 4:**
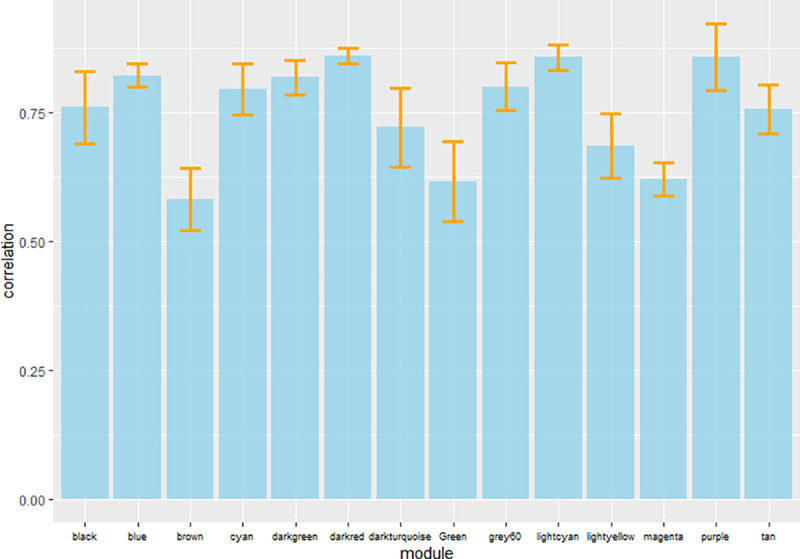
Bar graph of module stability. A graph of modules vs. connectivity correlation to assess the module stability. Connectivity correlation was obtained by random sampling of 50% of microarray samples for 1000 times. Error bars indicate Mean ± SD.

The microarray datasets (S1 Table and S2 Fig) used for the construction of co-expression network covers ~97% of *D*. *radiodurans* genome. All the data sets were from the same species and R1 strain of *D*. *radiodurans* to avoid species and strain related bias. The co-expression network was constructed by WGCNA in R package using default parameters [9], with few modifications as indicated in R script (S2.zip). The parameter β was selected as 6, which is the lowest possible power at which network topology is scale free (S3A Fig and S3B Fig). Further, the TOM based dissimilarity value was calculated based on adjacency coefficient and used as a distance measure (Fig 1). The dissimilarity measure obtained from TOM plot was used to define the cluster tree, the resulting dendrogram (Fig 2) thus obtained indicates highly interconnected subsets of genes called modules. Initially, 23 modules were obtained consisting of at least 20 genes per module. However, after merging the modules based on ME (if ME value > 0.8) 13 modules with a minimum of 20 genes each, were obtained (Fig 2). Modules thus obtained were namely ‘blue’, ‘black’, ‘brown’, ‘cyan’, ‘darkturquoise’, ‘green’, ‘lightcyan’, ‘lightyellow’, ‘purple’, ‘magenta’, ‘tan’, ‘darkgreen’ and ‘darkred’. Darkred was the smallest (38 genes) and blue was the largest (921 genes) module among others. The inter-modular connectivity based on ME showed that modules like darkgreen, brown, green and magenta have 6 connections, black and light cyan have 5 connections, cyan shows 4 connections, tan, blue, purple and darkturquoise showed 3 connections, whereas darkred as well as light yellow shows only 1 connection with another module (Fig 3). The random sampling of half of the microarray data from heterogeneous experiments showed that modules like brown, darkturquoise, green, lightyellow and magenta are less stable (Connectivity correlation < 0.7), whereas black, blue, cyan, darkgreen, darkred, lightcyan, purple and tan modules showed high stability (Connectivity correlation > 0.7) (Fig 4).

### Functional analysis of radiation and desiccation associated modules

The biological significance of a module was assessed by performing Gene ontology (GO) enrichment analysis. GO study revealed that out of 13 modules from the co-expression network, 11 showed significant functional enrichment (*p* value ≤ 0.05) for at least one or more ontology terms. The ontologies for radiation and desiccation associated modules are provided in Table 1. The biological functions of the modules obtained from the co-expression network are broadly categorized into metabolism, transport, DNA metabolism, gene expression, repair, regulation and modification, and RNA, Ribosome, protein metabolism and modification (S4 Fig).

**Table 1:** Gene ontology enrichment analysis.

Sr.No.	Module	Gene ontology category#	GOID	Number of genes in a module	Gene Ontology term	*p* value
1	Darkgreen	KEGG	KEGG:00400	251	Phenylalanine, tyrosine and tryptophan biosynthesis	1.53E-02
			KEGG:00270		Cysteine and methionine metabolism	1.87E-02
			KEGG:00362		Benzoate degradation	2.51E-02
		BP	GO:0016667		oxidoreductase activity, acting on a sulfur group of donors	3.81E-05
			GO:0000413		protein peptidyl-prolyl isomerization	1.53E-03
			GO:0016491		oxidoreductase activity	3.48E-03
			GO:0016209		antioxidant activity	1.53E-02
			GO:0034645		cellular macromolecule biosynthetic process	1.54E-02
			GO:1901701		cellular response to oxygen-containing compound	1.55E-02
			GO:0006790		sulfur compound metabolic process	1.84E-02
			GO:0070887		cellular response to chemical stimulus	1.84E-02
			GO:0006810		Transport	2.34E-02
			GO:0009055		electron transfer activity	2.68E-02
			GO:0008236		serine-type peptidase activity	3.37E-02
			GO:0051604		protein maturation	3.76E-02
			GO:0042592		homeostatic process	3.83E-02
			GO:0006284		base-excision repair	4.10E-02
			GO:0055076		transition metal ion homeostasis	4.10E-02
		MF	GO:0020037		heme binding	1.13E-02
2	Tan	KEGG	KEGG:02024	61	Quorum sensing	2.01E-02
		BP	GO:0004252		serine-type endopeptidase activity	1.08E-02
			GO:1901564		organonitrogen compound metabolic process	3.01E-02
			GO:0055086		nucleobase-containing small molecule metabolic process	3.52E-02
			GO:0072521		purine-containing compound metabolic process	4.06E-02
			GO:0044248		cellular catabolic process	4.38E-02
3	Cyan	KEGG	KEGG:00523	59	Polyketide sugar unit biosynthesis	1.28E-06
			KEGG:00521		Streptomycin biosynthesis	1.70E-05
			KEGG:03010		Ribosome	1.25E-02
		BP	GO:0009225		nucleotide-sugar metabolic process	6.15E-04
			GO:0005976		polysaccharide metabolic process	4.46E-03
			GO:0006518		peptide metabolic process	1.68E-02
			GO:1901135		carbohydrate derivative metabolic process	2.26E-02
			GO:0055086		nucleobase-containing small molecule metabolic process	2.54E-02
			GO:0016310		Phosphorylation	3.14E-02
			GO:0006412		Translation	3.69E-02
			GO:0004519		endonuclease activity	3.99E-02
		MF	GO:0003676		nucleic acid binding	4.79E-02
			GO:0016772		transferase activity, transferring phosphorus-containing groups	1.24E-03
			GO:0016462		pyrophosphatase activity	1.11E-02
			GO:0042623		ATPase activity, coupled	3.69E-02

#KEGG: Kyoto Encyclopedia of Genes and Genomes, BP: Biological process, MF: Molecular function

Darkgreen is the most interesting and important module (No. of genes: 251) as it showed association with both gamma radiation (*p* value = 0.006) and desiccation (*p* value = 0.00045) stress (S4 Table). This module showed enrichment of 3 KEGG pathways, 15 biological processes and 1 molecular function (Table 1 and S5A Fig). Amino acid metabolism and base excision repair are known to play a crucial role in the radiation stress adaptation of this microbe. Moreover, the transition of metal ion homeostasis, homeostatic process and transport identified in this study were also reported in previous condition (radiation) dependent network of *D*. *radiodurans* [18]. The darkgreen module was also found to be enriched with genes involved in oxidoreductase and antioxidant activity, which *D*. *radiodurans* is known to utilize under oxidative stress [45]. This module also showed the presence of some of the known radiation associated genes like DR_0070 (*ddrB*), DR_0326 (*ddr*D), DR_0326 (radiation-induced DNA repair protein), DR_0129 (DnaK), DR_0607 (GroEL), and DR_2494 (DNA-directed RNA polymerase subunit omega rpoB). Besides this, an important biological process was enriched in darkgreen module such as cellular response to chemical stimuli possess genes like DR_1279 (Mn family superoxide dismutase/Mn-SOD), DR_1982 (Thioredoxin reductase) and DR_A0259 (catalase). These genes are involved in defense against oxidative damage in *D*. *radiodurans* [3, 46]. However, pathway like benzoate degradation enriched in this module has not been studied before in the context of radiation or desiccation stress response of *D*. *radiodurans*.

Tan (No. of genes: 61) module also showed significant association with radiation (*p* value = 0.029) and desiccation (*p* value = 0.014) stress as that of darkgreen module (S4 Table). The Tan module was enriched with 1 KEGG pathway and 5 biological processes (Table 1 and S5B Fig). Quorum sensing pathway enriched in Tan module is known to enhance stress responses in various organisms like *V*. *cholerae* [47], *Pseudomonas aeruginosa* PAO1 [48, 49] as well as in *D*. *radiodurans* [50]. Besides this, the Tan module was also found to be enriched with the genes and proteins crucial for both gamma radiation and desiccation stress such as DR_0423 (Single- stranded DNA binding protein/DdrA) and DR_A0282. DdrA is known to protect 3’ DNA ends and also involved in maintaining genome integrity of *D*. *radiodurans* under gamma radiation stress [51]. Similarly, DR_A0282 was also known to be DNA binding protein and was found in a pool of nucleotide binding proteins during post irradiation recovery of *D*. *radiodurans* [52].

Interestingly darkgreen and Tan module which are found to be significant radiation, as well as desiccation responsive modules, shared some common functionality. The serine type endopeptidase activity or serine proteases were enriched in both darkgreen and tan module. Serine proteases are highly expressed after gamma radiation stress [21]. These serine proteases were found to be resistant to 6kGy of gamma radiation stress and also found to cleave oxidized proteins [3]. Both these modules also showed the presence of branched-chain amino acid ABC transporter genes (S5 Table). In *D*. *radiodurans*, ABC transporters are known to play an important role in the supply of exogenous amino acids as a backbone for peptides and proteins, befitting its proteolytic lifestyle [6]. Moreover, darkgreen and tan modules showed the presence of MutT/Nudix family protein (Nudix hydrolase). The Nudix hydrolases family protein is found in various organisms [53]. *D*. *radiodurans* is known to possess 25 Nudix hydrolases. In *D*. *radiodurans* it helps in maintaining the intracellular levels of nucleotide and also eliminates the damaged or oxidized nucleotides, thus play an important role in radiation stress [54].

Cyan (No. of genes: 59) is the smallest radiation responsive module (*p* value = 0.008) (S4 Table). The module was enriched with 3 KEGG pathways, 8 biological processes and 4 molecular functions (Table 1 and S5C Fig). Cyan module showed the presence of translational machinery such as ribosomal protein subunit (Large: L5, L14, L24, L29, and Small: S17). These were reportedly overexpressed during post irradiation recovery phase of *D*. *radiodurans* [21]. Besides, the cyan module also showed the presence of ParB an important chromosome partitioning protein found differentially expressed in response to gamma radiation stress [21] and its deletion imparted radiation sensitivity in *D*. *radiodurans* [55]. Furthermore, cyan module showed presence of ATP-dependent protease LA or Lon protease. The Lon proteases are two- component enzymes having proteolytic as well as ATPase activity. In *D*. *radiodurans* Lon protease are known to participate in removing or degrading the misfolded proteins, but their involvement in eliminating the radiation damaged protein is not known [21]. The cyan module also showed the presence of one of the crucial phosphorylation protein rqkA. The functionality of rqkA in phosphorylating DNA repair protein like PprA [56] and RecA [57] of *D*. *radiodurans* has been well documented and suggested the enhancement of DNA repair activity of both PprA and RecA in gamma radiation stress. Apart from these DNA repair proteins, the phosphorylation activity of rqkA has also been found to regulate the functional interaction of cell division proteins like FtsZ and FtsA in *D*. *radiodurans* [58]. However, no information was found with respect to the functionality of significant pathways enriched in cyan module like polyketide sugar unit and streptomycin biosynthesis for stress adaptation in *D*. *radiodurans*. The detailed lists of the genes mapped in radiation and desiccation modules can be found in S5 Table. Similarly, gene ontology analysis for remaining 8 modules was also carried out and detailed results are mentioned in S6 Table.

### Identification of important regulators and validation of their expression

**Fig 5:**
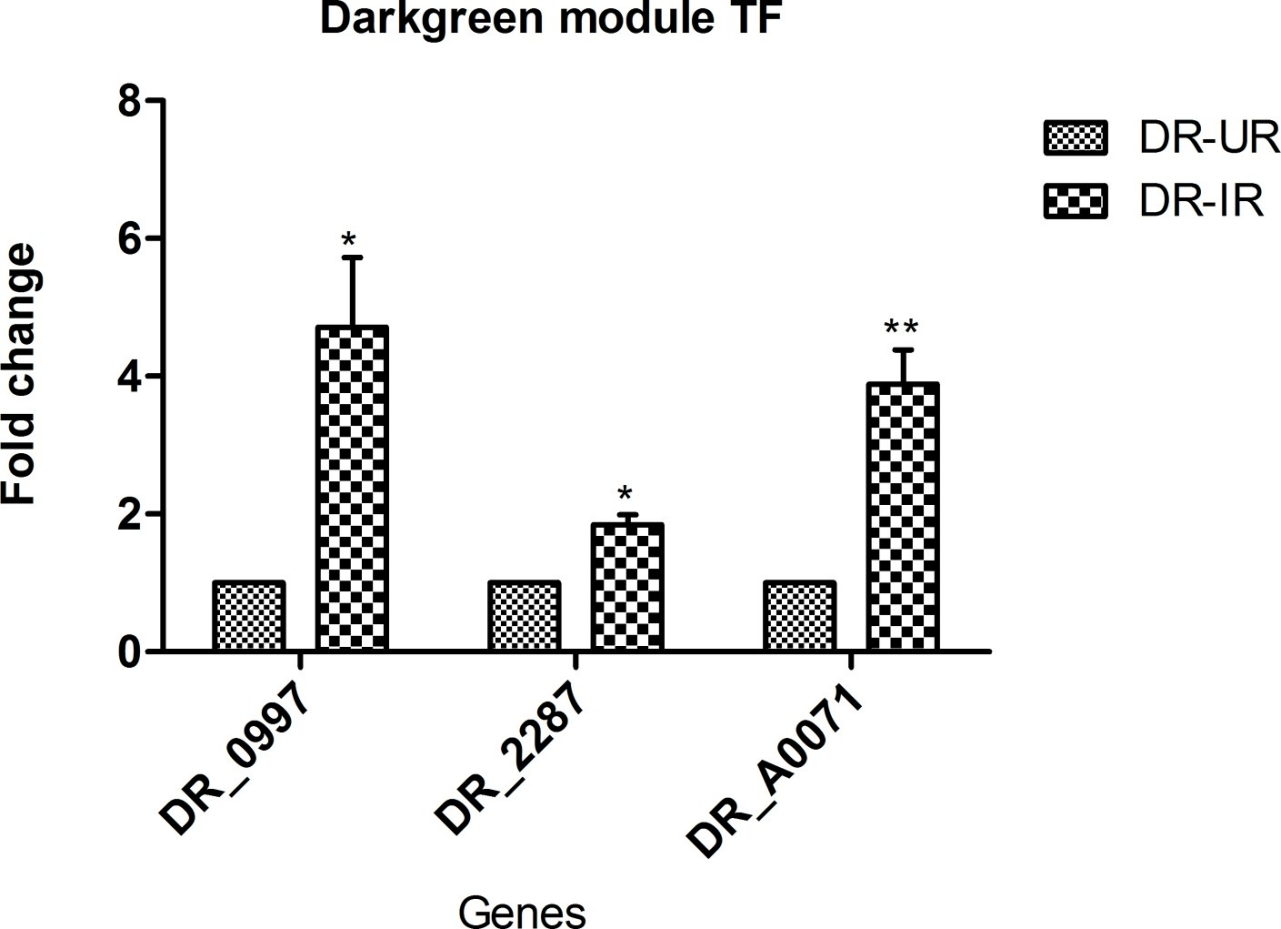
Bar graph of qRT-PCR of TFs in a radiation associated module. Expression profile of 3 TFs which are highly correlated to the hub gene in darkgreen module were checked at 90 minutes of PIR (Radiation dose—6 kGy) in WT *D*. *radiodurans*. Error bars indicate Mean ± SD. * *p* value ≤ 0.05, ** *p* value ≤ 0.01.

Transcription factors play an important role in the adaptation of bacteria in a hostile environment [59]. In our analysis, a total of 7 TFs were identified in association with radiation and desiccation stress responses, (S7 Table). However, the functional aspect for many of these TFs with respect to stress adaptation in *D*. *radiodurans* remains unexplored. In the present study, we exposed *D*. *radiodurans* to various doses (0–15 kGy) of gamma radiation stress. LD_50_ value for gamma radiation stress in *D*. *radiodurans* was found to be 9.3 kGy (S6 Fig). The expression profiles of 3 TFs, which are highly correlated to the hub gene in a darkgreen module, were confirmed by qRT-PCR in response to sub-lethal dose (6 kGy) of gamma radiation stress (Table 2 and Fig 5).

**Table 2:** Validation of transcription factors of modules.

Sr.No.	Module	Transcription factor	Fold change*	*p-*value*
1	Darkgreen	DR_0997	4	0.02
		DR_2287	1.8	0.01
		DR_A0071	3.8	0.009

*qRT-PCR fold change, *p* value by Student’s *t* test

It has been previously reported that TFs which are highly correlated with hub gene could be the potential regulators of respective module [34–36]. The regulation of darkgreen module appears to be complex as it possesses multiple TFs. These TFs showed presence of DNA binding/helix-turn-helix domain as well as other regulatory domains (S8 Table) as indicated by CDD analysis [32].

The radiation and desiccation associated darkgreen module showed enrichment of 6 TFs. Three out of six TFs viz DR_0997 (CRP/FNR family transcriptional regulator), DR_2287 (AsnC family transcriptional regulator) and DR_A00071 (transcriptional repressor SmtB) showed high correlation with the hub gene in darkgreen module (S7 Table). Genes associated with all enriched pathways, biological and molecular functions in the darkgreen module are found to be direct or close neighbors of these 3 TFs. Hence based on high correlation (> 0.8) of these TFs with the hub gene and associations with all other biological and molecular processes in darkgreen module, it can be speculated that these 3 TFs might be the potential regulators of darkgreen module. Expressions of DR_0997, DR_2287 and DR_A0071 were upregulated at 90 minutes of PIR (Table 2 and Fig 5) and have also been reported to be upregulated in response to 15 kGy of gamma irradiation [21] suggesting their possible role in radiation stress response.

CRP is a global regulator, known to regulate diverse functions in different prokaryotes [60] as well as in *D*. *radiodurans* [61]. In the present network the first neighbors of CRP, like DR_0070 (*ddrB*), DR_0326 (radiation-induced protein), DR_0349 (ATP-dependent protease LA), DR_1279 (*sodA*), and DR_A0006 (*cyclase*) were shown to be downregulated in CRP mutant of *D*. *radiodurans* [61]. Out of these, DR_0349 and DR_A0006 showed presence of motif TGTGA-N6-TCACA in the sequence upstream to respective ORFs [61]. Hence, CRP mutant data [61] and our network study further confirmed regulation of these two genes by CRP.

**Fig 6:**
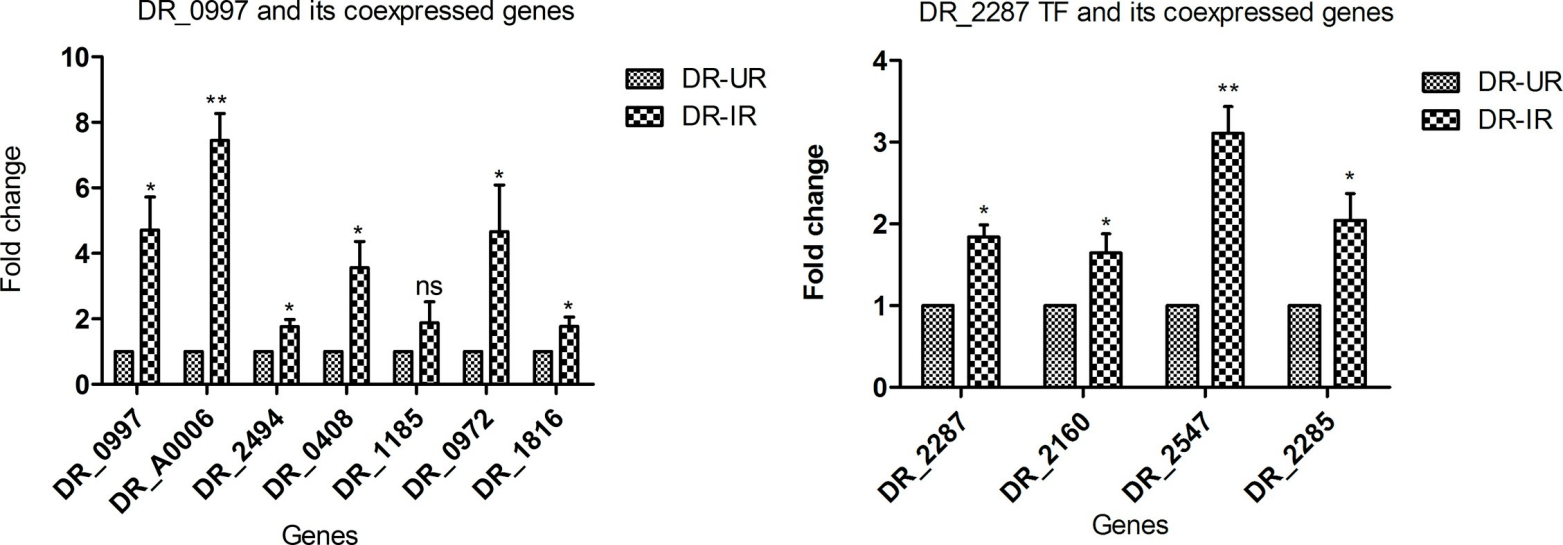
Bar graph of qRT-PCR of selected TFs and their first neighbors in radiation stress associated module. Expression profiles of direct or close neighbors of TF (A) DR_0997 and (B) DR_2287 in a darkgreen module at 90 minutes of PIR (Radiation dose-6 kGy) in WT *D*. *radiodurans*. Error bars indicate Mean ± SD. * *p* value ≤ 0.05, ** *p* value ≤ 0.01.

Moreover, we also found that around 79% of first neighbors of CRP (i.e. 198 out of 251 first neighbors) (S9 Table) showed differential expression in response to gamma radiation stress [21]. Amongst them we have validated the expression of 6 first neighbors of CRP in response to gamma radiation stress (Table 3 and Fig 6A). Recently, Meyer et al., 2018 have predicted 33 targets for CRP based on presence of CRP binding motif in the promoter sequences of the targets [62]. Of these predicted targets, 6 targets are enriched as first neighbors of CRP in darkgreen module. Taken together, these results confirm the regulatory role of CRP under gamma radiation stress.

**Table 3:** Validation of co-expressed genes with transcription factors in a darkgreen module.

Module	Transcription factor	Co-expressed genes	Fold change*	*p* value*
Darkgreen	DR_0997	DR_2494	1.7	0.02
		DR_0408	3.5	0.03
		DR_0972	4.6	0.04
		DR_A0006	7.2	0.005
		DR_1816	1.7	0.04
		DR_1185	1.88	ns
	DR_2287	DR_2285	2.0	0.03
		DR_2547	3.0	0.008
		DR_2160	1.6	0.04

* qRT-PCR fold change, *p* value by Student’s *t* test, ns = non-significant

The AsnC family transcriptional regulator is found in both bacteria and archaea. It is involved in the regulation of amino acid metabolism, ATP biosynthesis as well as in bacterial pathogenesis [63]. *Deinococcus-Thermus group* has acquired AsnC family transcriptional regulator through horizontal gene transfer [64] but its role in *D*. *radiodurans* is less explored. Also, all genes enriched in various processes and pathways in darkgreen module are direct or close neighbors of AsnC family transcriptional regulator. Furthermore, we found that out of 251 first neighbors of AsnC family transcriptional regulator 198 genes showed differential expression (S10 Table) in previous gamma radiation stress experiment [21]. Amongst these, we have also validated the expression of 3 first neighbors in response to gamma radiation stress (Table 3 and Fig 6B). These genes are DR_2285 (A/G, A/C and A/GO specific adenine glycosylase) involved in base excision repair [3], DR_2547/hemA (glutamyl-tRNA reductase) and DR_2160 (porphobilinogen synthase) involved in porphyrin and chlorophyll metabolism which leads to the synthesis of tetrapyroll [65]. The porphyrin molecule is protective against reactive oxygen species [66] generated by oxidative or radiation stress. Hence, our data suggest the possible regulatory role of AsnC family transcriptional regulator under gamma radiation stress.

Moreover, the darkgreen module genes enriched in various ontology process are close neighbors of DR_A0071 (SmtB family transcriptional repressor) as that of DR_0997 and DR_2287, thus speculating important role of this TF in stress adaptive pathways apart from DR_0997 and DR_2287. SmtB family transcriptional repressor is known to maintain metal ion homeostasis in prokaryotes [67]. Its expression was significantly upregulated in response to cadmium stress in *D*. *radiodurans* [43]. Our results and previous data [21, 42] showed increased expression of DR_A0071 in response to gamma radiation stress in *D*. *radiodurans*.

The remaining 3 TFs in darkgreen module like DR_1204 (4-carboxymuconolactone decarboxylase), DR_A0128 (hypothetical protein), and DR_1628 (transcriptional activator TipA) showed low-moderate correlation (0.5–0.7) with the hub gene. CDD analysis of all of these TFs showed the presence of helix-turn helix domain, however, the functionality of these TFs under radiation stress is unexplored.

Tan module possesses TetR family transcriptional regulator (DR_B0126) which showed a correlation of 0.78 with the hub gene (S7 Table). Genes associated with quorum sensing and other biological/molecular processes in tan module are close neighbors of DR_B0126. Hence this TF may be regulating the functionality (Table 1) of Tan module.

### Hub gene Identification and its functional annotation

In the co-expression network, higher the connectivity, more important is the gene in the module and such genes are termed as hub gene [17]. Because of high intramodular connectivity, functional annotation of a hub gene in a module is crucial. The most connected hub genes were screened and thus, we obtained 13 hub genes, one for each module and amongst them, 6 genes were annotated with hypothetical function (S11 Table). Functional uniformity in a coexpressed module allows us to assign potential biological functions or gene ontology to hypothetical hub genes [8]. Hence, the gene ontology or functional analysis of a module in which these genes are enriched helped us unravel the functionality of 5 hypothetical hub genes (S12 Table). The 5 hub genes are assigned to darkgreen (DR_2251), green (DR_1095), lightcyan (DR_0110), lightyellow (DR_1461) and magenta (DR_2185). As described earlier [8], the functionality of close neighbors (TOM > 0.01) of hub genes can be assigned to the respective hub genes of the module. In the present study, we have obtained enrichment of multiple GO terms/functionality for close neighbors of hub genes. Hence, highly significant or top GO term/functionality was assigned to the respective hypothetical hub gene in a module. Therefore, based on TOM > 0.01 (for close neighbors) and *p* < 0.05 (for GO terms) these hypothetical hub genes are predicted to be involved in biological functions in *D*. *radiodurans* such as DR_2251 (oxidoreductase activity, acting on a sulfur group of donors), DR_1095 (Glycine, serine and threonine metabolism), DR_0110 (oxidoreductase activity), DR_1461 (zinc ion binding), and DR_2185 (ribonucleoside bisphosphate metabolic process) (S12 Table). Moreover, these predictions need an extensive investigation to prove direct involvement of hypothetical hub gene in *D*. *radiodurans* biology.

## Study limitations

Our co-expression network analyses have few limitations to mention in particular. The prominent drawback of our coexpression network study is that it is constructed using a limited number of microarray datasets (total experiments = 9, total datasets = 61). Scarcity of microarray data limits the construction of a robust co-expression network. We constructed an unsigned network in this study with the motivation to extract gene pairs that are co-expressed (both positive as well as negative associations) [68] irrespective of the experimental conditions, to discern potential stress responsive gene regulators if any. However, our un-signed co-expression network may carry potential positive correlation bias [19]. Also, except for DR_0997 (CRP) [61], TF binding site information is unknown for other TFs identified in the present study associated with stress response.

## Conclusion

This study is the first of its kind, based on condition independent co-expression network in *D*. *radiodurans*. Various radiation and desiccation stress related pathways have been explored by genome-wide transcriptomics and proteomics approaches. We have utilized the systems biology approach to predict potential stress responsive pathways, biological and molecular functions as well as regulators. Based on our analyses we speculate that transcription factors like DR_0997, DR_2287 and DR_A0071 could potentially be involved in the regulation of radiation as well as dessciation stress responsive pathways and functions in *D*. *radiodurans*. Our analyses provide novel leads that need to be investigated further to fully elucidate extreme DNA damage resistance of this organism. Additionally, we observed that the pathways like benzoate degradation, polyketide sugar unit biosynthesis and streptomycin biosynthesis may also be important in stress response of *D*. *radiodurans*. Furthermore, we have utilized this coexpression network for assigning the gene ontology or biological function to hypothetical hub genes. Hence our analysis has envisaged various leads worth exploring further.

## Supporting information

S1 Fig Batch effect visualization. (A) Box plot: The figure shows overall box plot of all experiments grouped in to 9 batches (9 different experiments, a-i), x-axis shows microarray samples grouped into batches (a-i), whereas y-axis is the gene expression values (B) and MDS plot: The plot shows that 9 different experiments grouped in to batches a to i, indicated by different colors and form a single cluster. The x and y axis shows Coordinate 1 and 2 respectively.
(TIF)

S2 Fig Distribution of microarray experiments. Microarray experiments used in the current study are broadly categorized in to four groups Cadmium, Nacl, MMC stress, gene knockout and radiation.
(TIF)

S3 Fig Determining the value of β parameter in WGCNA. Parameter β was used in adjacency matrix formula (AdjMat_ij_ = |S_ij_|^β^). The value of β was obtained by scale-free topology criterion. To obtain smooth average connectivity of the network we selected value of β = 6 using graph such as (A) scale independence and (B) mean connectivity.
(TIF)

S4 Fig Overall gene ontology of all modules. Pie chart that represents broad categorization of gene ontology terms of all 11 modules carried out by ClueGO a cytoscape plugin.
(TIF)

S5 Fig Pie chart of gene ontology terms in stress associated modules. Pie chart shows percent of gene ontology terms in radiation and desiccation responsive modules (A) Darkgreen, (B) Tan and (C) Cyan
(TIF)

S6 Fig Graph of LD_50_ value for *D. radiodurans* R1. Graph depicting the LD_50_ for WT *D. radiodurans* when subjected to 0–15 kGy of gamma radiation stress. Error bar indicates Mean ± SD.
(TIF)

S1 Data The zip file consists of detail dataframe of all microarray samples for batch effect visualization, module generation and stability analysis. It also consists of list of transcription factors, and genome annotation file which are used in current analysis.
(ZIP)

S2 Data The zip file consists of R script used for box plot, generation of modules and module stability analysis. (ZIP)

S1 Table Detailed list of microarray experiments. (XLS)

S2 Table List of oligo-nucleotides used for qRT-PCR. (XLS)

S3 Table Microarray experiment description and outcome of the respective study. (XLS)

S4 Table Screening of radiation and desiccation responsive modules. Total number of genes in all modules and their respective *p* value.
(XLS)

S5 Table Detail list of DEGs and DEPs in radiation and desiccation (Darkgreen and Tan) and radiation (cyan) responsive module. (XLS)

S6 Table Detailed list of gene ontology terms for all remaining 8 coexpressed modules. (XLS)

S7 Table Biweight mid correlation between TF and module hub gene. (XLS)

S8 Table Detailed domain analysis by CDD of all TFs enriched in darkgreen and tan module. (XLS)

S9 Table Average gene expression values of DR_0997 first neighbors matched with previous transcriptome data. Gene expression values depicted in red are statistically significant.0-24 hours are the PIR time points.
(XLS)

S10 Table Average gene expression values of DR_2287 first neighbors matched with previous transcriptome data. Gene expression values depicted in red are statistically significant. 0–24 hours are the PIR time points.
(XLS)

S11 Table List of hub genes in all modules. (XLS)

S12 Table Functional analysis of close neighbors (TOM > 0.01) of hypothetical hub genes in a module. (XLS)
