# Supplementary figures and images for: Construction, analysis and validation of co-expression network to understand stress adaptation in Deinococcus radiodurans R1
Source: PLoS One. 2020 Jun 24;15(6):e0234721. doi: 10.1371/journal.pone.0234721 (PMC7314050; doi:10.1371/journal.pone.0234721)

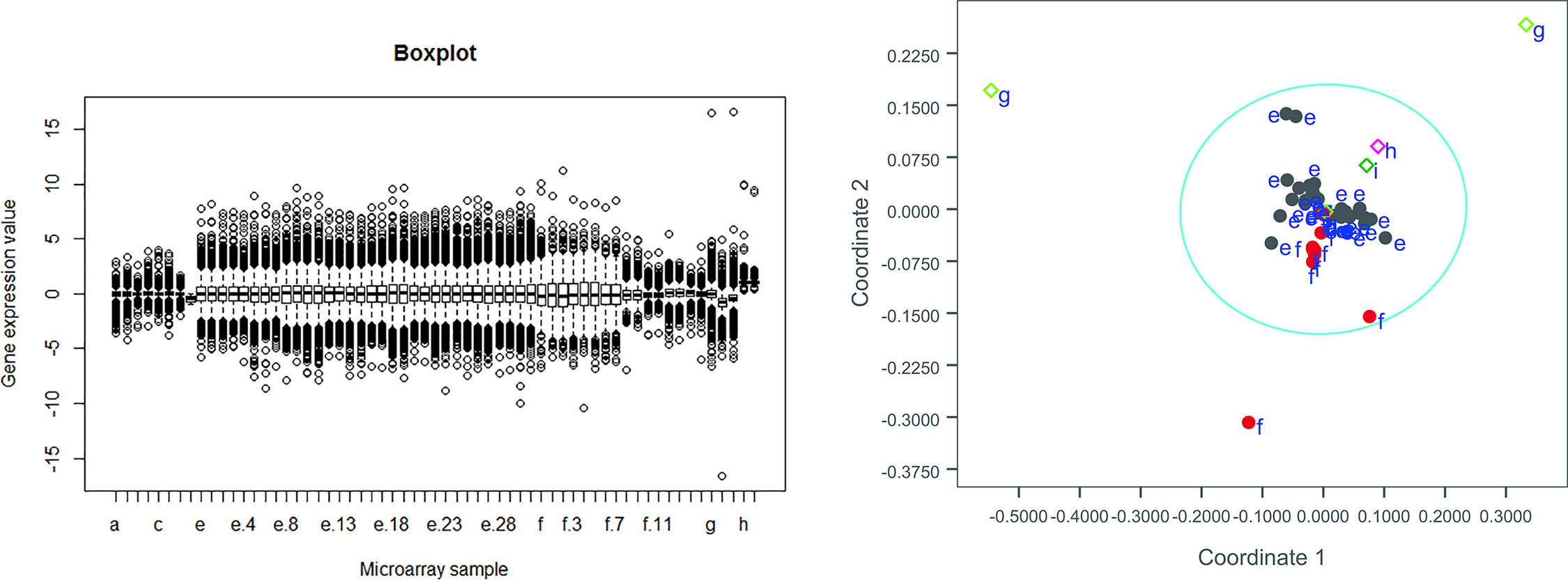

Supplement: S1 Fig — (A) Box plot: The figure shows overall box plot of all experiments grouped in to 9 batches (9 different experiments, a-i), x-axis shows microarray samples grouped into batches (a-i), whereas y-axis is the gene expression values (B) and MDS plot: The plot shows that 9 different experiments grouped in to batches a to i, indicated by different colors and form a single cluster. The x and y axis shows Coordinate 1 and 2 respectively. (TIF) [file pone.0234721.s001.tif]

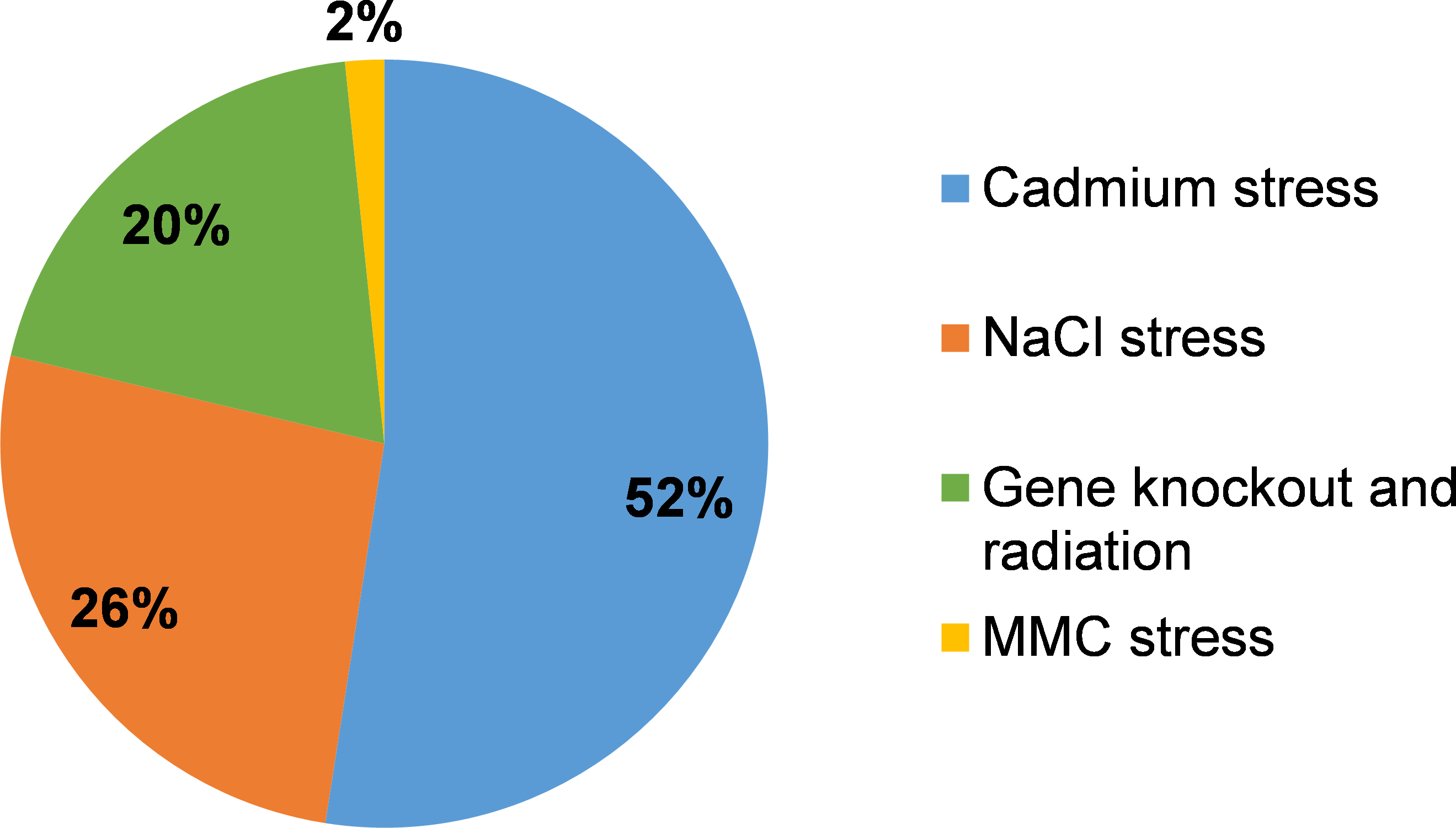

Supplement: S2 Fig — Microarray experiments used in the current study are broadly categorized in to four groups Cadmium, Nacl, MMC stress, gene knockout and radiation. (TIF) [file pone.0234721.s002.tif]

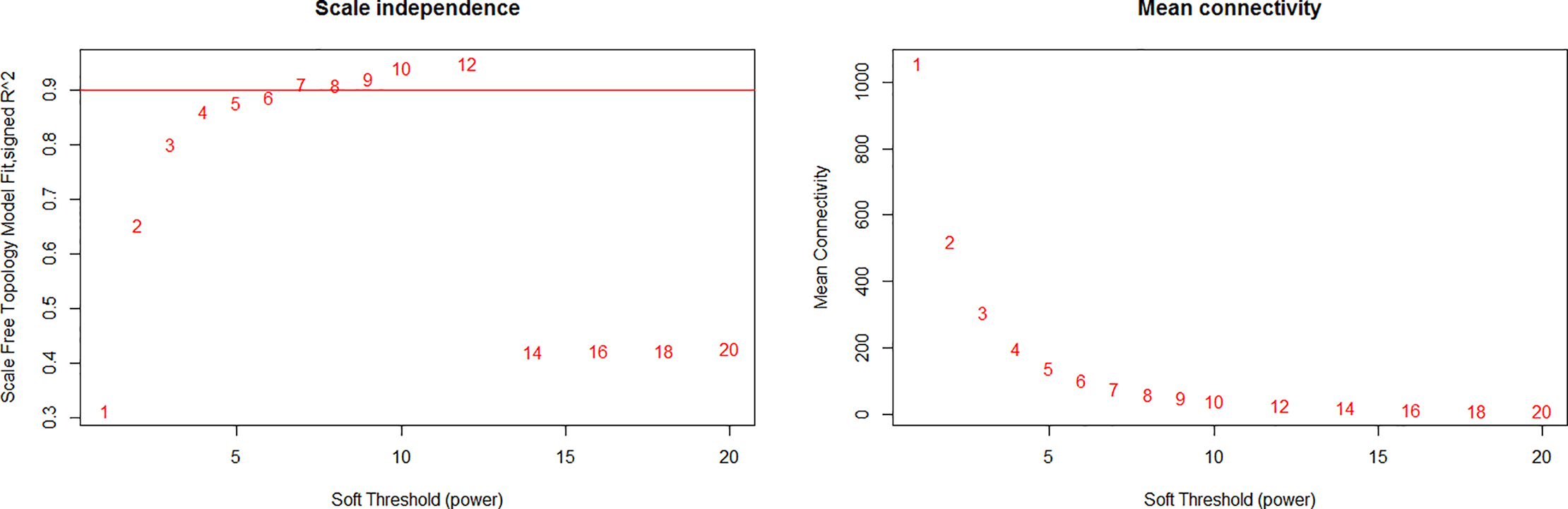

Supplement: S3 Fig — Parameter β was used in adjacency matrix formula (AdjMatij = |Sij|β). The value of β was obtained by scale-free topology criterion. To obtain smooth average connectivity of the network we selected value of β = 6 using graph such as (A) scale independence and (B) mean connectivity. (TIF) [file pone.0234721.s003.tif]

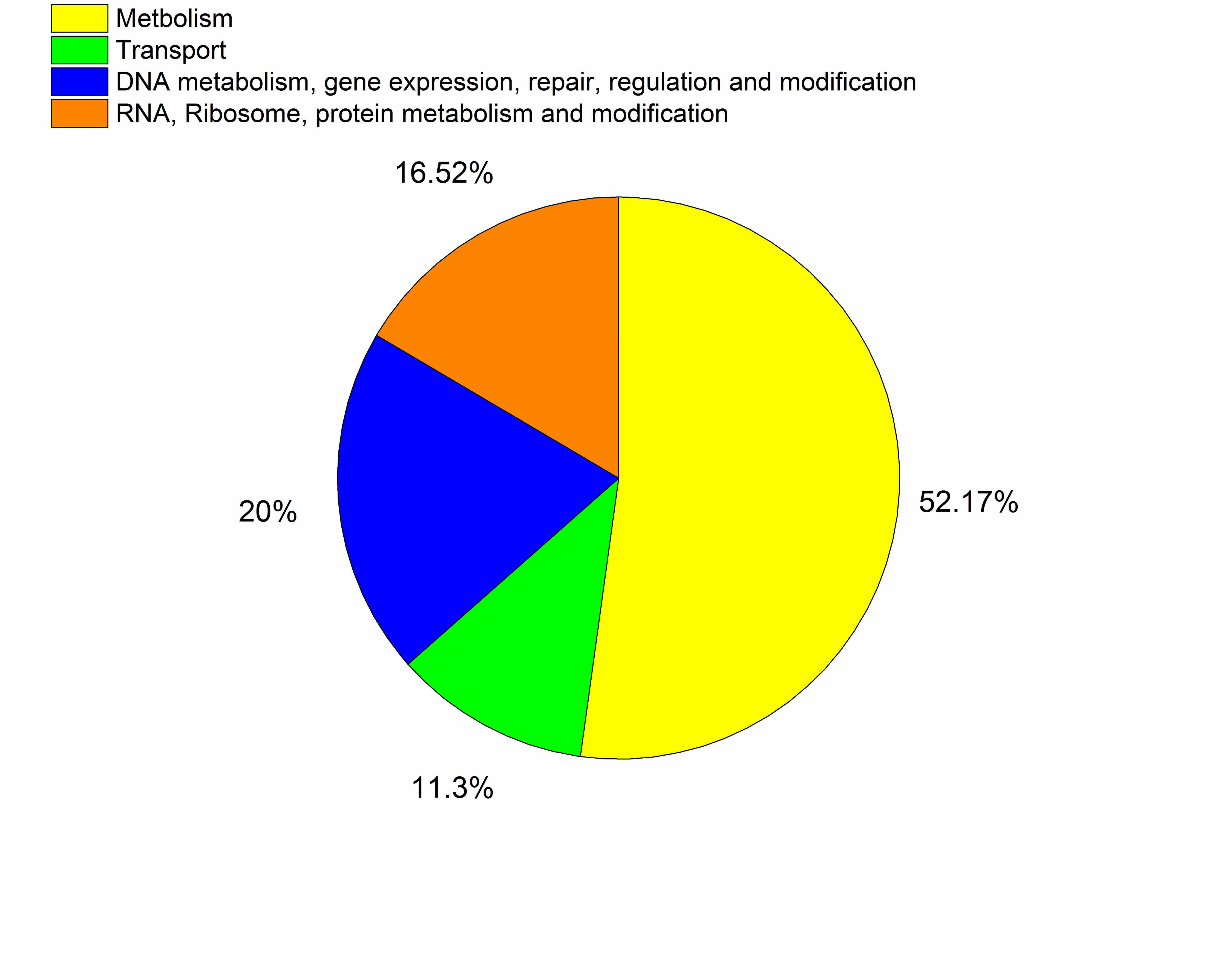

Supplement: S4 Fig — Pie chart that represents broad categorization of gene ontology terms of all 11 modules carried out by ClueGO a cytoscape plugin. (TIF) [file pone.0234721.s004.tif]

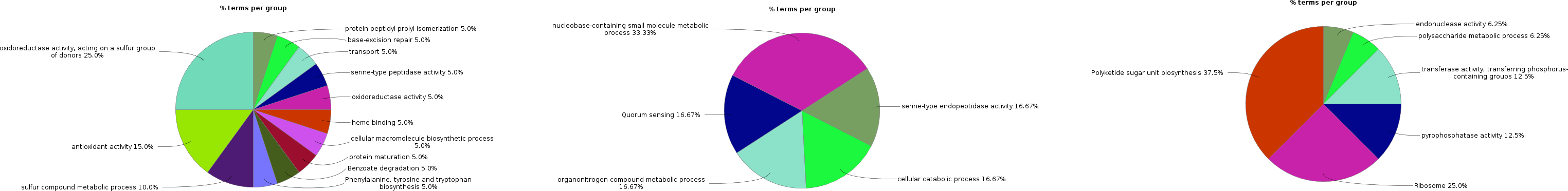

Supplement: S5 Fig — Pie chart shows percent of gene ontology terms in radiation and desiccation responsive modules (A) Darkgreen, (B) Tan and (C) Cyan (TIF) [file pone.0234721.s005.tif]

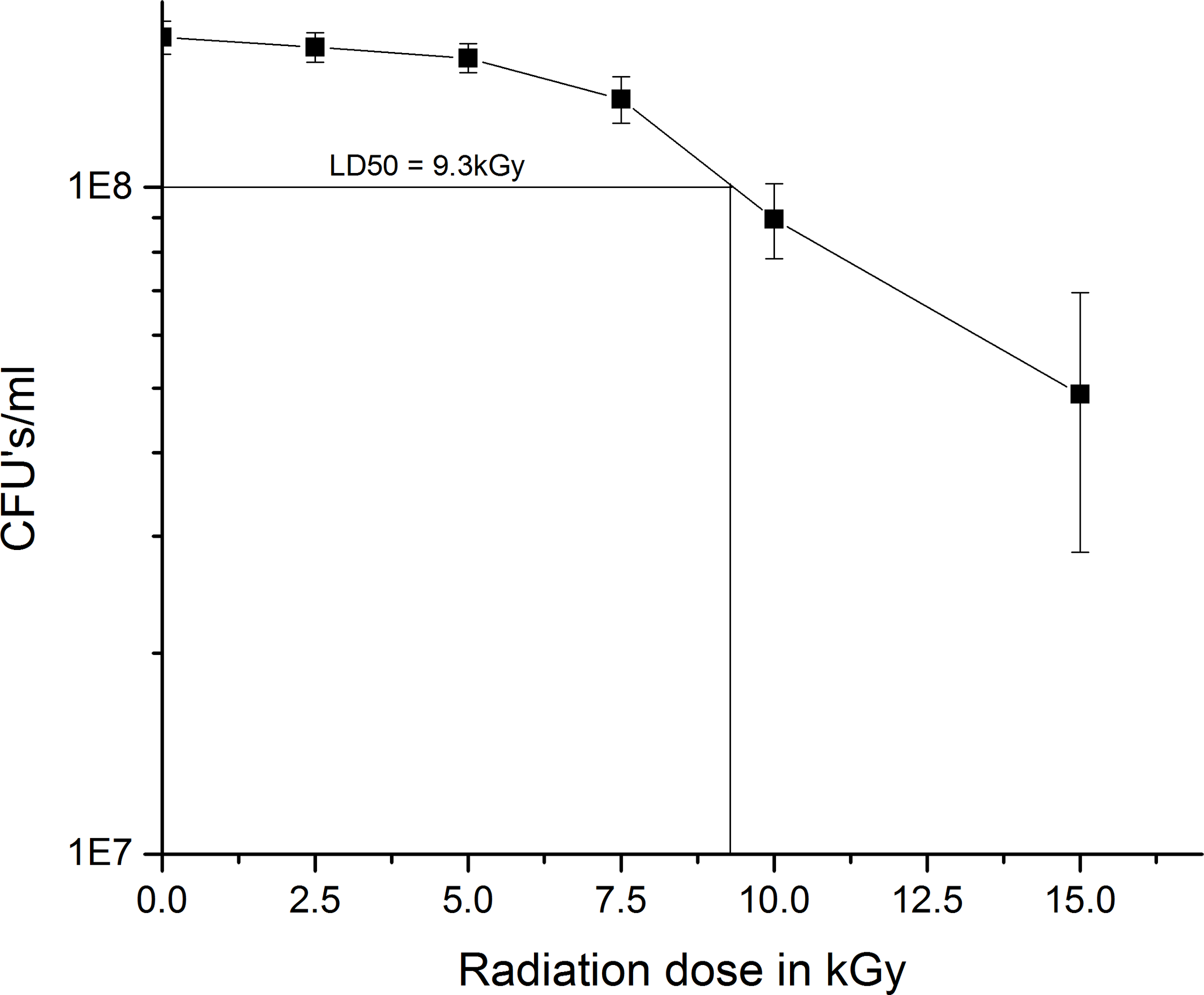

Supplement: S6 Fig — Graph depicting the LD50 for WT D. radiodurans when subjected to 0–15 kGy of gamma radiation stress. Error bar indicates Mean ± SD. (TIF) [file pone.0234721.s006.tif]
